# Proanthocyanidins polymeric tannin from *Stryphnodendron adstringens* are active against *Candida albicans* biofilms

**DOI:** 10.1186/s12906-015-0597-4

**Published:** 2015-03-19

**Authors:** Raul Leal Faria Luiz, Taissa Vieira Machado Vila, João Carlos Palazzo de Mello, Celso Vataru Nakamura, Sonia Rozental, Kelly Ishida

**Affiliations:** Laboratório de Biologia Celular de Fungos, Instituto de Biofísica Carlos Chagas Filho, Universidade Federal do Rio de Janeiro, Rio de Janeiro, RJ Brazil; Laboratório de Biologia Farmacêutica, Palafito, Departamento de Farmácia Universidade Estadual de Maringá, PR, Brazil; Laboratório de Inovação Tecnológica no Desenvolvimento de Fármacos e Cosméticos, Departamento de Ciências Básicas da Saúde, Universidade Estadual de Maringá, PR, Brazil; Laboratório de Quimioterapia Antifúngica, Departamento de Microbiologia, Instituto de Ciências Biomédicas, Universidade de São Paulo, Av. Prof. Lineu Prestes 1374, 05508-900 SP, Brazil

**Keywords:** Antifungal, Biofilm, *Candida albicans*, Proanthocyanidin, *Stryphnodendron adstringens*, Tannins

## Abstract

**Background:**

Biofilm formation is important in *Candida albicans* pathogenesis and constitutes a mechanism of antifungal resistance. Thus, we evaluated the effect of proanthocyanidins polymer-rich fractions from *Stryphnodendron adstringens* (fraction F2 and subfraction F2.4) against *C. albicans* biofilms.

**Methods:**

Firstly, the antifungal activity of F2 and F2.4 against planktonic cells of *Candida albicans* (ATCC 10231) was determined using broth microdilution method. Anti-biofilm effect of F2 and F2.4 was evaluated during biofilm formation or on mature biofilm of *C. albicans* and compared with standard antifungals amphotericin B and fluconazole. Metabolic activity of sessile and dispersion cells from biofilms after antifungal treatments were measured using a tetrazolium reduction assay and the biofilm total biomass was quantified by crystal violet-based assay. Morphological alterations after treatments were observed using scanning electron microscopy.

**Results:**

The anti-biofilm effect of F2 and F2.4 were comparable to standard antifungals (amphotericin B and fluconazole). F2 and F2.4 treatments reduced biofilm metabolic activity (in sessile and in dispersion cells) during biofilm formation, and in mature biofilms, unlike fluconazole, which only prevents the biofilm formation. Treatments with F2, F2.4 or fluconazole reduced biofilm biomass during biofilm formation, but not in mature biofilm. Amphotericin B presented higher inhibitory effect on biofilm formation and on mature biofilm of *C. albicans*. F2 and F2.4 treatments led to the appearance of dumbbell-shaped blastoconidia and of blastoconidia clusters in biofilms.

**Conclusion:**

Proanthocyanidins polymer-rich fractions from *S. adstringens* successfully inhibited *C. albicans* planktonic growth and biofilm development, and they represent a potential new agent for the treatment of biofilm-associated candidiasis.

## Background

*Candida* spp. are commensal yeasts that, in healthy individuals, colonize mainly mucosal surfaces of the oral cavity, and the gastrointestinal and urogenital tracts, without causing disease symptoms. However, these fungi may cause opportunistic infections in humans with certain physiological disorders, or in patients who have become immunocompromised or are undergoing therapy with broad-spectrum antibiotics. About 96% of all opportunistic mycoses are caused by *Candida* sp. [1], and *C. albicans* is still the major pathogenic species worldwide, causing 90% of vulvovaginal candidiasis [2] and 50 – 70% of invasive candidiasis cases [3], with a mortality rate of 30–40% [3].

In Brazil, *Stryphnodendron adstringens* (Mart.) Coville (Leguminosae) is one of the most frequently used medicinal plants in the treatment of vaginal infections and wounds, and it is also used as astringent, anti-diarrhoeal, antimicrobial or hypoglycaemic agent [4]. The stem bark of *S. adstringens* is rich in tannins (10-37%) [4], representing mainly flavan-3-ols and proanthocyanidins, such as prodelphinidins and prorobinetinidins [5-7]. Previous pharmacological studies have demonstrated that tannins extracted from *S. adstringens* stem bark have healing properties [8], analgesic and anti-inflammatory activity [9] and gastric anti-ulcerogenic effects [10]. Several studies have also demonstrated that tannins from *S. adstringens* have inhibitory activity against parasitic protists [11-13], viruses [14] and bacteria [9,15].

Previous reports from our group have demonstrated the antifungal activity of fractions derived from *S. adstringens* stem bark (fraction F2 and subfraction F2.4) against planktonic cells (in suspension) from vaginal isolates of *Candida* spp. [7], and against *Cryptococcus neoformans* [16]. Treatment with the proanthocyanidin polymeric tannins present in subfraction F2.4 (hexameric polymer with monomeric units of prodelphinidins, prorobinetinidins and gallic acid residues with molecular weight 2,114 Da.) also altered some virulence factors of *C. albicans* as well as led to alterations in budding and on cell wall morphology [7]. In addition, toxicological studies using rodents have also reported low side effects after treatment with proantocyanidin polymer-rich fraction (F2) [17,18].

Biofilms are defined as heterogeneous microbial communities of cells adhering to an abiotic or biotic surface, while embedded in a polymeric extracellular matrix (produced by biofilm cells). In biofilms, microbial cells display altered phenotypes in comparison to those observed in planktonic cells of the same species and strain, mainly in their increased resistance to antifungal treatments [19,20]. *C. albicans* biofilms are resistant to a variety of clinical antifungal agents, including amphotericin B and fluconazole [19,20], the major antifungal agents used for the treatment of both superficial and invasive candidiasis [21].

The development of new molecules with activity against *C. albicans* biofilms is paramount to add to the limited therapeutic options currently available for the effective treatment of *Candida* infections involving biofilm formation. Previously, we showed that *S. adstringens* fraction F2 and subfraction F2.4 are active against *Candida albicans* planktonic cells [7]. In this work, we extend these findings by demonstrating that fraction F2 and subfraction F2.4 from *S. adstringens* are also effective against *C. albicans* biofilms, and against dispersion biofilm cells, which are important for disease dissemination inside the host.

## Methods

### Microorganism

Antifungal effects were tested against *Candida albicans* ATCC 10231. This strain was maintained in Sabouraud dextrose agar (Becton, Dickinson and Company, Sparks, USA) at 4°C and subcultured twice in the same medium at 35°C, for 24 h before each assay.

### Extraction

*Stryphnodendron adstringens* stem bark was collected in São Jerônimo da Serra, Paraná, Brazil, in March 2010. A voucher specimen was deposited at the Herbarium of the Universidade Estadual de Maringá (HUEM #14321), and it was identified by Prof. Dr. Cássia Mônica Sakuragui (Federal University of Rio de Janeiro). The stem bark was dried at room temperature and pulverized. Fraction F2 and subfraction F2.4 were prepared as described by Ishida and co-workers [7], and kept as lyophilized extracts at -20°C in small aliquots. As previously reported, subfraction F2.4 consists of a hexameric tannin composed of monomeric units of prodelphinidins and prorobinetinidins, and gallic acid residues (molecular weight, 2,114 Da) [7]. The method of fractions preparation was validated by Costa et al. [22], and the subfraction 2.4 was identified by solid-state nuclear magnetic resonance (NMR) spectrometry using a Varian spectrometer (Mercury Plus 300 [7.02T]) operating at 75 MHz for ^13^C and 300 MHz for ^1^H. The identities of the polymeric tannin in subfraction F2.4 were confirmed using ^13^C NMR, by comparison of the spectrum obtained here with results reported by Ishida et al. [7].

### Antifungal agents

Two antifungal were used as references: fluconazole (FLC; Pfizer, São Paulo, Brazil), diluted in water (2,000 μg/mL) and amphotericin B (AMB; Sigma Chemical Co., USA), diluted in DMSO (1,600 μg/mL) and stored at -20°C.

### Antifungal susceptibility tests on planktonic cells

The antifungal susceptibility of *C. albicans* ATCC 10231 planktonic cells was assessed by broth microdilution assay as described in Document M27-A3 [23]. The concentrations tested were: 0.48 - 250 μg/mL, for fraction F2 or subfraction F2.4; 0.03 - 16 μg/mL AMB; and 0.125 - 64 μg/mL FLC. The minimum inhibitory concentration (MIC) value is defined as the lowest concentration that inhibit 50% of fungal growth (IC_50_, for azoles) or 90% of fungal growth (IC_90_, for polyenes) determined by visual inspection or spectrophotometric reading at 492 nm in a microtitre plate reader (SpectraMAX 340 Tunable Microplate Reader, Molecular Devices Ltd, USA) [23]. Here, we also considered the IC_50_ value to be the MIC for the fraction F2 and subfraction F2.4 from *S. adstringens*.

The MFC is defined as the lowest concentration of a given compound that fails to produce fungal growth, and was determined by transferring an aliquot of fungal samples treated with concentrations higher than the MIC into fresh Sabouraud dextrose agar plates, which were then incubated at 35°C for 48 h. Fungicidal effects were considered significant when the MFC value was ≤ 4 × MIC value. Above this value, the antifungal effect was considered fungistatic [24].

### Treatment of biofilms with antifungal agents

To evaluate the effect of antifungals on biofilm formation, we used a protocol modified from previously described assays for *C. albicans* biofilm formation and antifungal treatment [25,26]. Aliquots of 100 μL of *C. albicans* ATCC 10231 yeast suspensions (1 × 10^7^ CFU/mL, in RPMI 1640 medium buffered with 0.16 MOPS and supplemented with 2% glucose) were transferred into each well of polystyrene, flat-bottomed 96-well plates, and incubated at 35°C under constant agitation for 1.5 h. Then, supernatants were gently aspirated to remove any remaining non-adhering cells, and 100 μL of fresh medium with or without antifungal agents were added to the wells.

The choice of antifungal agent concentrations to be tested against biofilms was based on MIC values obtained for the treatment of planktonic cells. The following concentrations of antifungal agents were tested: 0.5, 2, 8 and 32 μg/mL of AMB; 2, 8, 32 and 64 μg/mL of FLC; and 31.25, 125, 500 and 1,000 μg/mL of fraction F2 or subfraction F2.4. After the addition of antifungal agents, plates were incubated for 24 or 48 h at 35°C under constant agitation.

To evaluate the susceptibility of mature biofilms to antifungal agents, biofilm formation was allowed to proceed for 24 h at 35°C. After mature biofilms were formed, the supernatant was removed, 100 μL of fresh medium with or without antifungal agents (in the same concentrations described above) were added to the wells, and the plates were incubated for a further 24- or 48-h period, at 35°C and under constant agitation.

After biofilm formation in the presence of the antifungals or mature biofilm treatment, the supernatant containing the dispersive cells was collected in eppendorf tubes for future analysis and the biofilm was quantified using two different approaches.

Metabolic activities of sessile and dispersion cells from *C. albicans* biofilms after antifungal treatments (during biofilm formation or on mature biofilm) were measured using a tetrazolium reduction (XTT) assay [25]; and the biofilm total biomass was quantified by crystal violet-based assays [27].

### Scanning electron microscopy (SEM)

*C. albicans* ATCC 10231 biofilms were formed over 5-mm sections of central venous catheters (CVCs), as previously described [28], in the presence or absence of antifungal agents at concentrations corresponding to 64 × MIC (32 μg/mL AMB, 1,000 μg/mL fraction F2 or subfraction F2.4). Antifungal treatments were also performed on mature biofilms. Catheters containing biofilms were fixed in 2.5% glutaraldehyde and 4% formaldehyde, in 0.1 M cacodylate buffer, for 1 h at room temperature, and post-fixed in 1% osmium tetroxide and 1.25% potassium ferrocyanide (in 0.1 M cacodylate buffer) for 30 min. The samples were dehydrated in ethanol increasing concentrations, critical-point-dried in CO_2_, coated with gold and observed in a FEI-Quanta 250 scanning electron microscope (FEI, Japan).

### Statistical analyses

Statistical analyses were performed on the PRISM 5.0 software (Graphpad, USA), using the Dunnett’s test (one-way analysis of variance). Statistical significance was accepted when p < 0.05.

## Results and discussion

The main objective of the present work was to evaluate the effect of fractions F2 and subfraction F2.4 (proanthocyanidins polimeric tannins) from *S. adstringens* against *C. albicans* biofilm formation and mature biofilms (previously formed for 24h before drug treatment). *S. adstringens* was selected for this work because it is commonly used in the treatment of vaginal infections in Brazil, where *C. albicans* is the main fungal pathogenic species [2].

Given that biofilm cells are notoriously more resistant to antifungal therapy than planktonic (suspension) cells [19,20,29], we evaluated the inhibitory activity of *S. adstringens* fractions on *C. albicans* ATCC 10231 biofilms using concentrations of these fractions higher than the MIC values determined on planktonic cells. Kuhn and co-workers [30] reported that anti-biofilm activity is achieved at considerably higher drug concentrations (8-32 and 32-1,024 fold, for AMB and FLC, respectively) than those used for planktonic cell growth inhibition. Considering that fraction F2 and subfraction F2.4 behaved similarly to FLC and AMB when tested against planktonic cells, we defined that our analysis should cover concentrations up to 64 fold the MIC value for planktonic cells, which represented the higher limit of the therapeutic range of these fractions.

*C. albicans* ATCC 10231 planktonic cells were susceptible to treatment with fraction F2 and subfraction F2.4 (MIC values of 15.6 μg/mL for both). The MIC values obtained for *C. albicans* ATCC 10231 were slightly higher than those previously observed by our group for other isolates of *Candida* spp. (MIC values between 0.97 and 7.80 μg/mL) [7]. For comparison, MIC values for FLC (1 μg/mL) and AMB (0.5 μg/mL) were also determined in parallel. MFC values revealed that only AMB had a fungicidal effect, whereas FLC, F2 and F2.4 had fungistatic effect against planktonic cells (MFC values of 1 μg/mL for AMB, 64 μg/mL for FLC, and 250 μg/mL for both F2 and F2.4).

**Figure 1:**
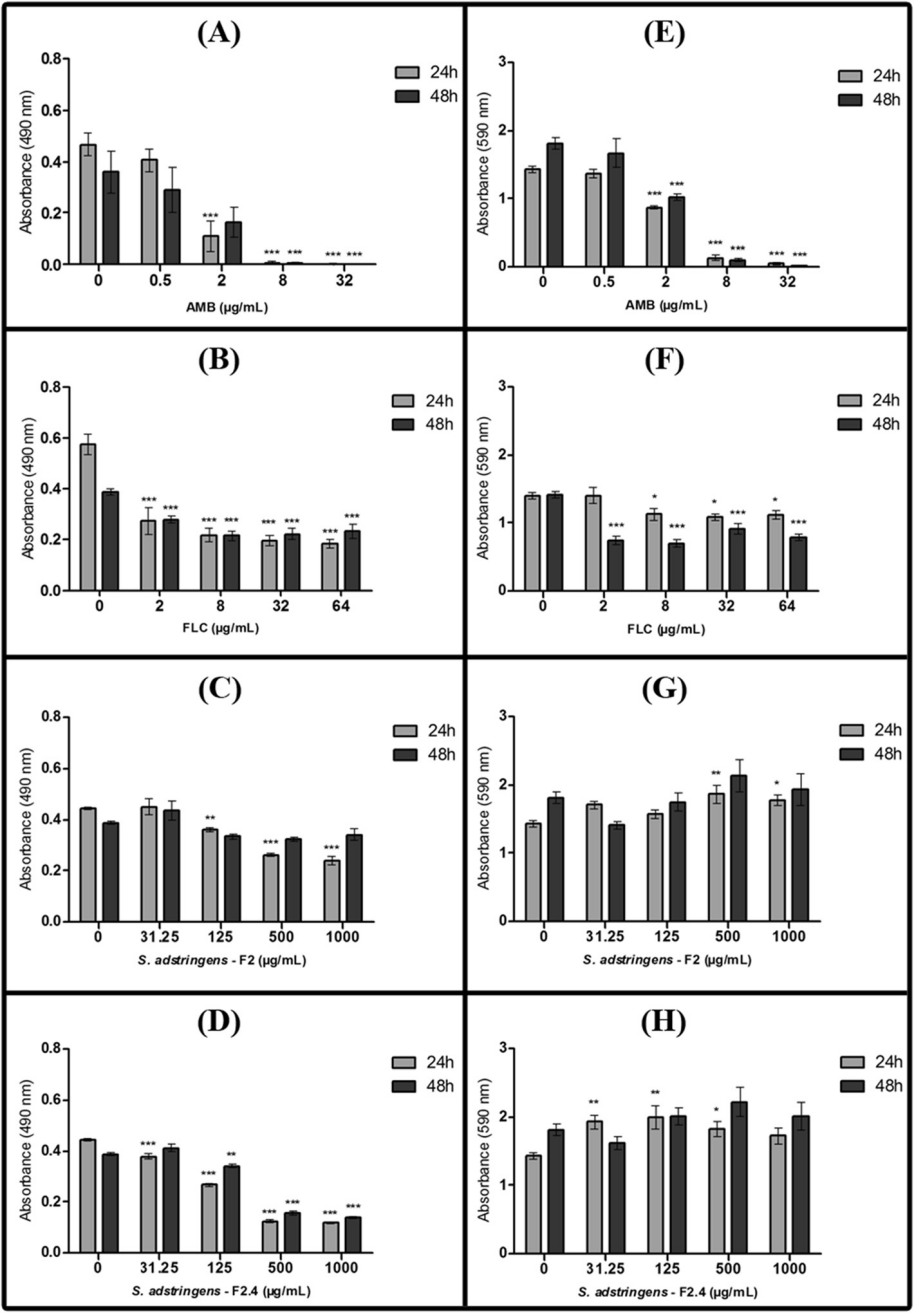
**Effect of fraction F2 and of subfraction F2.4 from** ***Stryphnodendron adstringens*** **on the biofilm formation of** ***Candida albicans*** **ATCC 10231.** The metabolic activity of biofilm sessile cells was quantified using an XTT assay **(A–D)** and total biofilm biomass was quantified using a cristal violet-based assay **(E–H)**, after growth in the presence of amphotericin B **(A** and **E)**, fluconazole **(B** and **F)**, F2 **(C** and **G)** or F2.4 **(D** and **H)**, for 24 or 48h, at 35°C, and under agitation. The error bar represents the standard error of the mean. *p < 0.05; **p < 0.01; ***p < 0.0001 (compared to untreated biofilms, using the Dunnett’s test).

**Figure 2:**
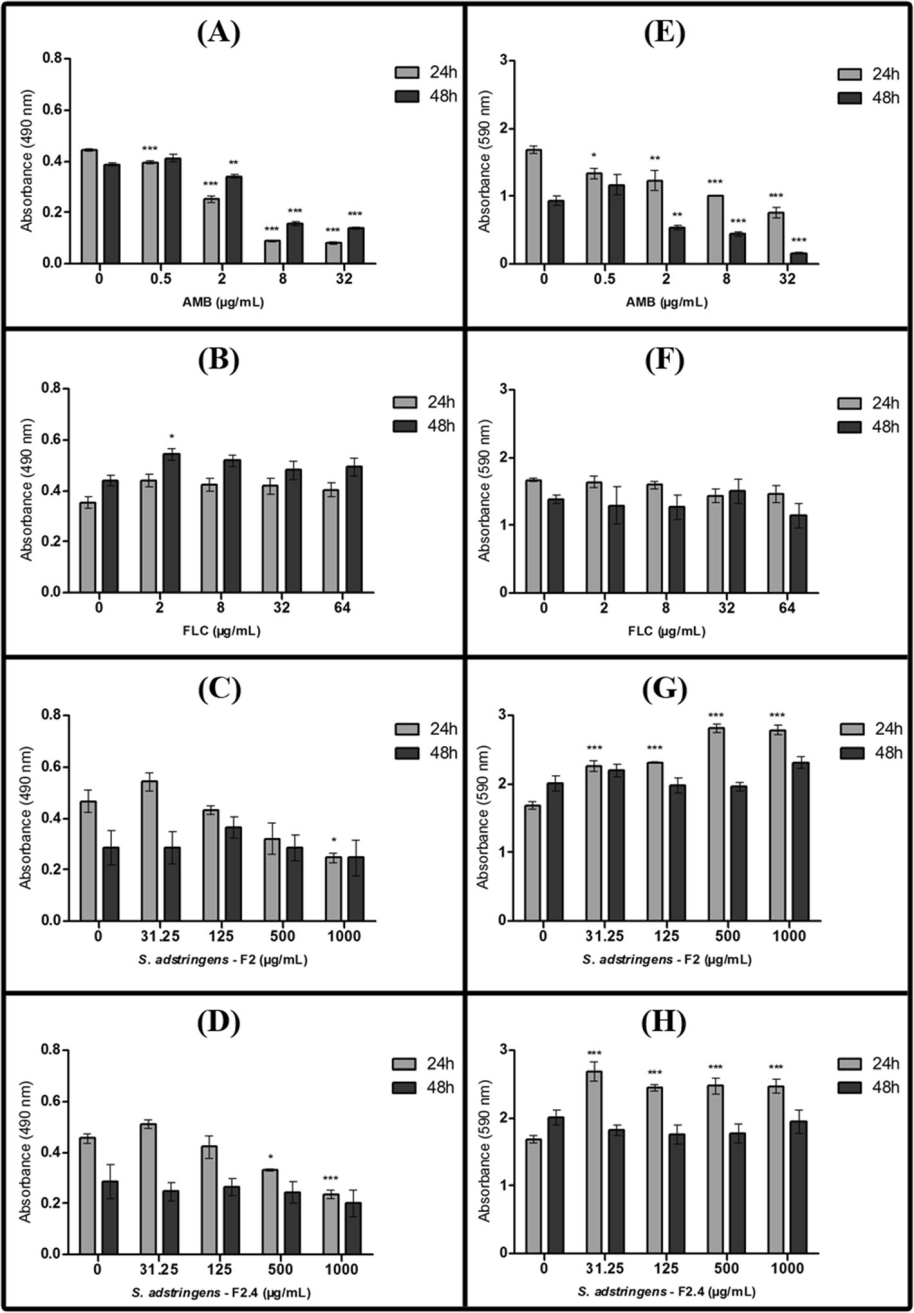
**Effect of fraction F2 and of subfraction F2.4 from** ***Stryphnodendron adstringens*** **on mature biofilms of** ***Candida albicans*** **ATCC 10231.** The metabolic activity of biofilm sessile cells was quantified using an XTT assay **(A–D)**, and the total biofilm biomass was quantified using a cristal violet-based assay **(E–H)**. Biofilms formed for 24h at 35°C were treated for 24 or 48h, at 35°C (and under agitation) with amphotericin B **(A** and **E)**, fluconazole **(B** and **F)**, F2 **(C** and **G)** or F2.4 **(D** and **H)**. The error bar represents the standard error of the mean. *p < 0.05; **p < 0.01; ***p < 0.0001 (compared to untreated biofilms, using the Dunnett’s test).

**Figure 3:**
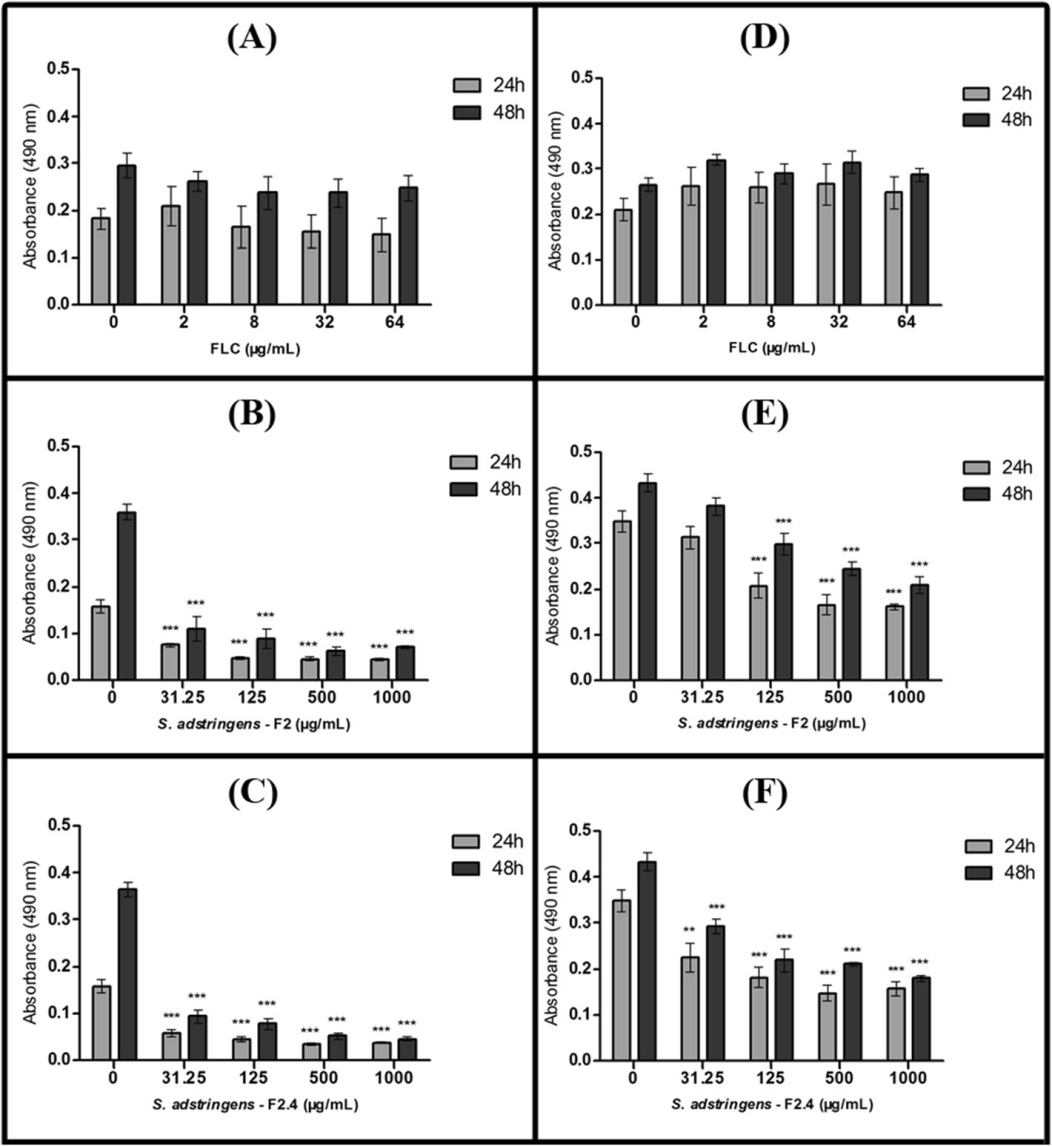
**Effect of fraction F2 and of subfraction F2.4 from** ***Stryphnodendron adstringens*** **on dispersion cells from biofilms of** ***Candida albicans*** **ATCC 10231.** The metabolic activity of dispersion cells was estimated using an XTT assay, using dispersion cells collected from biofilms under formation **(A – C)** or mature (formed for 24h at 35°C, prior to the start of treatment) **(D – F)**. Biofilms were treated for 24 or 48h at 35°C with fluconazole **(A** and **D)**, F2 **(B** and **E)** or F2.4 **(C** and **F)**. Dispersion cells from biofilms treated with amphotericin B were not analyzed, since these were found in insufficient numbers to allow accurate quantification. The error bar represents the standard error of the mean. **p < 0.01; ***p < 0.0001 (compared to untreated biofilms, using the Dunnett’s test).

*S. adstringens* fractions presented an inhibitory activity against *C. albicans* ATCC 10231 biofilms formation comparable to the standard antifungals (AMB and FLC) (Figure 1). F2 and F2.4 were also active against mature biofilm sessile cells (Figure 2), and against dispersed cells from biofilm (Figure 3). On the other hand, the standard antifungal FLC, which prevents biofilm formation, does not affect the metabolic activity of sessile cells from mature biofilm, neither the spread of infection by biofilm dispersion cells (Figures 1, 2 and 3).

All antifungal tested here caused a dose-dependent decrease in the metabolic activity of biofilm sessile cells with a highest antifungal activity observed after 24 h of treatment, during biofilm formation (Figure 1A-D). AMB was the most active antifungal and concentrations as low as 2 μg/mL (4 × MIC) of this drug were able to inhibit up to 78% and 57% of biofilm sessile cells metabolism at 24 h and 48 h, respectively. Total inhibition of biofilm sessile cells metabolism (98% at 24 h of incubation) was observed at concentration ≥ 8 μg/mL (≥16 × MIC) of AMB (Figure 1A). FLC inhibited 52% of biofilm sessile cells metabolism after 24 h or 48 h, when 2 μg/mL (2 × MIC) were used, and only 41% of biofilm sessile cells metabolism after 48 h of incubation, using the maximum concentration of 64 μg/mL (64 × MIC) during biofilm formation (Figure 1B).

Among the *S. adstringens* fractions tested here, subfraction F2.4 significantly inhibited biofilm sessile cells metabolism at concentrations ≥ 31.25 μg/mL (2 × MIC) (p < 0.05) (Figure 1D). However, the greatest effect was observed at concentration ≥ 500 μg/mL (≥32 × MIC), when 72% inhibition of biofilm sessile cells metabolism was observed after 24 h of incubation, and 60% inhibition after 48 h (Figure 1D). The effect of fraction F2 on the metabolism of biofilm sessile cells was less pronounced after 24h of biofilm formation (up to 47% inhibition at 1,000 μg/mL), and no significant inhibitory effect was observed after 48 h of incubation (Figure 1C). Further, metabolic activity quantification revealed that subfraction F2.4 was more effective at inhibiting biofilm formation than fraction F2 (Figures 1C and D).

The total biofilm biomass after treatment with antifungal during biofilm formation, including both viable and non-viable cells was quantified using a CV staining assay (Figures 1E-H). Despite the reduction in biofilm metabolic activity after treatment with F2 and F2.4, there was no significant change in the total biofilm biomass even after 48 h of incubation with the highest concentrations of these fractions. However, AMB treatment resulted in a dose-dependent reduction of the total biofilm biomass, with complete inhibition of biofilm formation at concentrations ≥ 8 μg/mL (≥16 × MIC) (p < 0.001), comparing to the untreated group (Figure 1E). Also, a reduction in the biofilm biomass of 32-50% was observed with FLC treatment at concentrations ≥ 2 μg/mL (≥2 × MIC) after 48 h of incubation (p < 0.001; Figure 1F).

Mature biofilms of *C. albicans* ATCC 10231 were less susceptible to AMB than biofilms under development (Figure 2A). Treatment with 2 μg/mL (4 × MIC) AMB resulted in an inhibition of 44% and 13% of biofilm sessile cells metabolic activity, after 24 h and 48 h of incubation, respectively (Figure 2A). When 32 μg/mL (64 × MIC) of AMB was used, reductions of 82% and 65% in sessile cells metabolism were achieved, after 24 h and 48 h of treatment, respectively (Figure 2A). Although FLC treatment resulted in higher inhibition of biofilm formation compared to fractions from *S. adstringens* (Figure 1B-D), FLC had no significant effect on mature biofilms at any of the concentrations tested (Figure 2B), while treatment of mature biofilms for 24 h with 1,000 μg/mL (64 × MIC) of fraction F2 or of subfraction F2.4 reduced by 47% and 51% the metabolic activity of mature biofilm sessile cells, respectively (Figure 2C and D).

Treatment of mature biofilms with AMB resulted in a dose-dependent reduction in biofilm biomass (Figure 2E), matching the corresponding reduction in biofilm metabolic activity after treatment (Figure 2A). AMB induced a prominent reduction of mature biofilm biomass (37-80%) at concentrations ≥ 2 μg/mL (≥4 × MIC; p < 0.001), after 48 h of incubation, comparing to the untreated group (Figure 2E). The total biomass of mature biofilms was not reduced after treatment with FLC, fraction F2 or subfraction F2.4 at concentrations up to 64 × MIC after both 24 and 48 h incubation periods (Figure 2F-H), despite the decrease in metabolic activity observed on biofilm cells after treatment with F2 and F2.4 (Figure 2C and D). In addition, the total biomass of mature *C. albicans* biofilms increased in comparison with the untreated group after 24 h of incubation with F2 or F2.4 (Figure 2G and H). We hypothesized that this could be happening for two reasons: (i) metabolically inactive cells remain attached to biofilms upon drug treatment, contributing to maintain biofilm biomass; and/or (ii) the extracellular matrix (ECM) of biofilms increases during treatment, as a protective mechanism against proantocyanidin polymeric tannins from *S. adstringens*.

In *C. albicans* biofilms, the ECM can prevent antifungal agent penetration into the innermost biofilm layers [20], acting as a physical barrier to protect cells embedded in the biofilm community from the access of antimicrobial agents, thus increasing biofilm drug resistance. The efficiency of the ECM as an ‘obstacle’ to drug penetration seems to depend on the amount and nature of the matrix, as well as on the physicochemical properties of the antifungal drug [20]. Further, the ECM quantification assays performed on treated biofilms should clarify the importance (if any) of increased ECM production in biofilm biomass maintenance during treatment with FLC, F2 or F2.4.

Fractions F2 and subfraction F2.4 from *S. adstringens* were more effective at inhibiting biofilm formation, but these fractions were also active against mature biofilms. Still, both fraction F2 and subfraction F2.4 were more effective against mature biofilms than FLC, which did not affect pre-formed biofilms (Figure 2A-D). Biofilms are considerably more resistant to antifungal drugs than planktonic cells, and this statement is particularly true for azoles (such as FLC) [19,29]. Indeed, despite the poor effectiveness of FLC against biofilms, this drug is the first option for the treatment of superficial and mucosal *Candida* infections [29]. Although fractions F2 and F2.4 were overall less effective than AMB against *C. albicans*, previous works showed that extracts from *S. adstringens* are less toxic to red blood cells and to epithelial cells than polyene agents (such as AMB) [13]. Still, doses used in this work were safely within the therapeutic window reported for fraction F2 in rodent models [17,18]. Absence of F2 genotoxicity to mice at concentrations as high as 2,250 mg/kg were also reported [17]. Lethal dose of 3,015 mg/kg [18] and chronic treatment for 90 days with 100-200 mg/kg did not affect blood biochemical parameters or tissue morphology as demonstrated by Costa et al. [18].

During biofilm development, cells that become detached from biofilms are known as ‘dispersion cells’ [31]. These cells are particularly important for disease progression, because they may colonize other sites in the host, expanding the infection or, in the worst case scenario, starting a disseminated infection [31]. This cells display distinct virulence properties when compared to their planktonic counterparts, including enhanced adherence, filamentation, biofilm formation, and increased pathogenicity in a murine model of hematogenously disseminated candidiasis [31]. The dispersion cells from biofilms were also analysed in this work (except for those from AMB treated samples, where biofilm growth was too insipid to yield a sufficient number of dispersion cells for analysis). Dispersion cells from biofilm formation or from mature biofilms displayed a dose-dependent reduction in metabolic activity after treatment with *S. adstringens* fractions, and this effect was more pronounced for the subfraction F2.4 than for fraction F2 (Figure 3B-F). Dispersion cells from biofilms formed in the presence of *S. adstringens* fractions at concentrations ≥ 31.25 μg/mL (≥2 × MIC) had significantly reduced metabolic activity in comparison to those released from untreated biofilms after 24 h or 48 h of treatment (fraction F2, 49-83% of inhibition; subfraction F2.4, 61-89% of inhibition) (Figure 3B and C). Both fractions yielded metabolic activity inhibition in dispersion cells above 50%, as early as 24 h after the start of treatment on mature biofilm at concentrations ≥ 500 μg/mL (≥32 × MIC) (p < 0.001) (Figure 3E and F). Moreover, F2 and F2.4 were significantly more effective at reducing the metabolic activity of dispersion cells from biofilms than treatment with FLC, where dispersion cell metabolic activity was similar to that observed in the untreated group (Figure 3A and D). These exciting results suggest that treatment with proantocyanidins polymer-rich fractions from *S. adstringens* stem bark might be able to prevent the dissemination of *C. albicans* infections, due to its effect on metabolic activity of dispersion cells.

**Figure 4:**
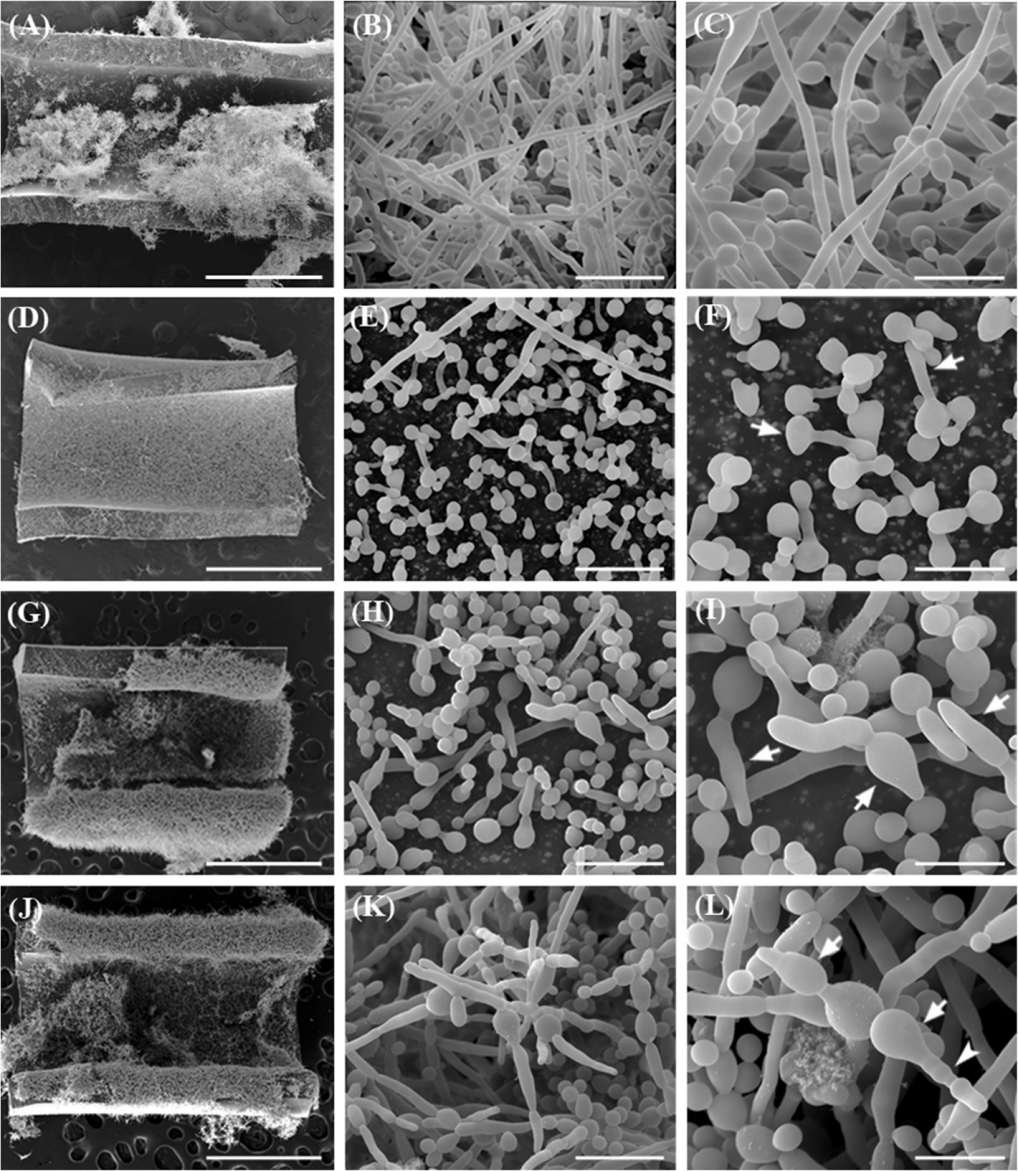
**Scanning electron microscopy of** ***Candida albicans*** **ATCC 10231 biofilms formed in the presence of fraction F2 or subfraction F2.4 from** ***Stryphnodendron adstringens*** **.** Biofilms were treated with antifungal agents concentrations 64 fold higher than the minimum inhibitory concentration (MIC) values determined for the treatment of planktonic cells for 24-h incubations at 35°C. Compared to untreated controls **(A-C)**, biofilms formed in the presence of 32 μg/mL amphotericin B **(D-F)** displayed a reduction in fungal biomass, with filamentation inhibition and the presence of irregular buds (arrows in F). Treatment with 1,000 μg/mL of fraction F2 **(G-I)** or subfraction F2.4 **(J-L)** led to the formation of biofilms with blastoconidia cells of altered shape, including ‘dumbell’ cells (I and L, arrows) and those with elongated bud necks (L, arrowhead). Scale bars: 1 mm **(A, D, G** and **J)**, 20 μm **(B, E, H** and **K)** and 10 μm **(C, F, I** and **L)**.

**Figure 5:**
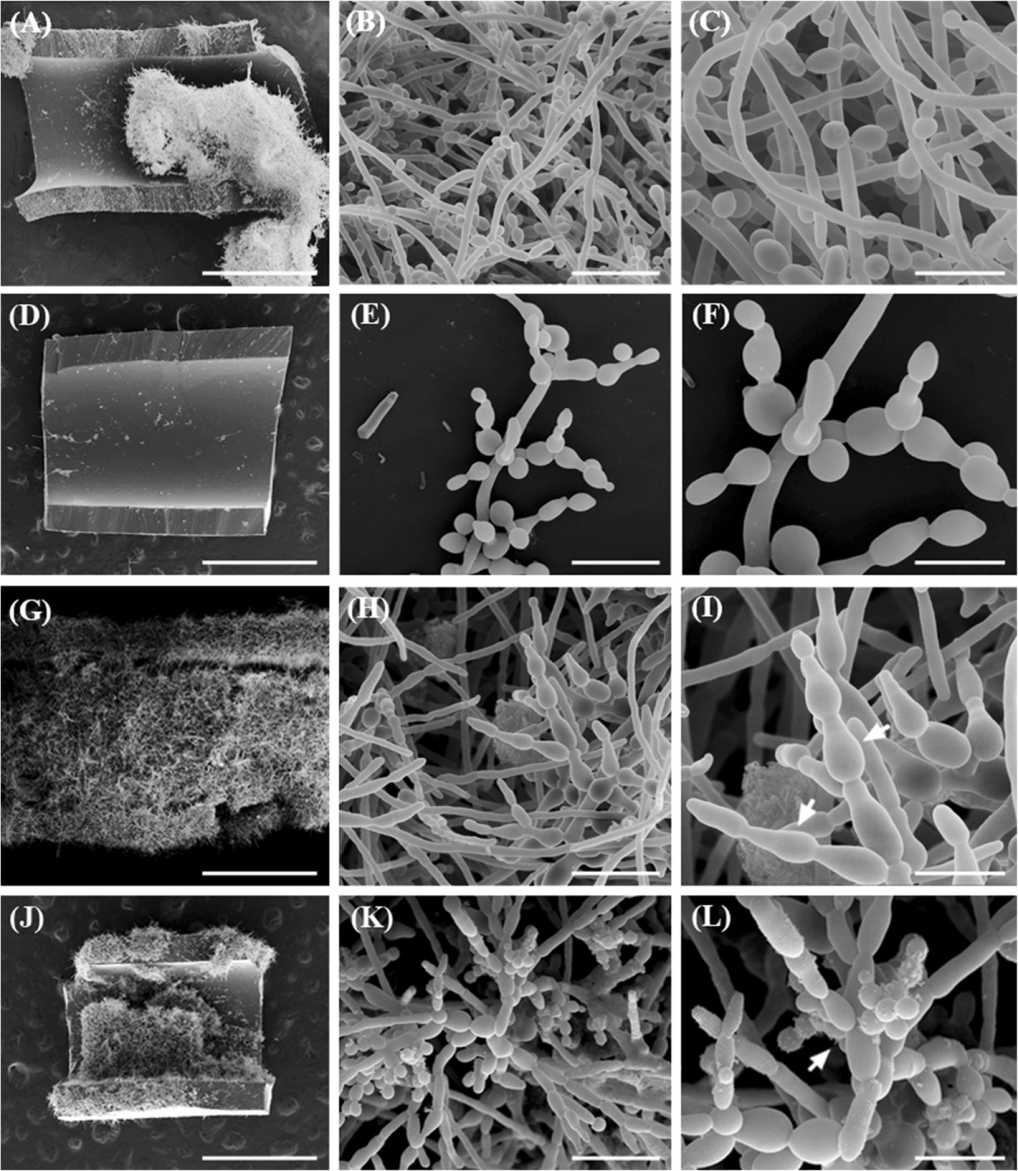
**Scanning electron microscopy of mature biofilms from** ***Candida albicans*** **ATCC 10231 treated with fraction F2 or subfraction F2.4 from** ***Stryphnodendron adstringens*** **.** Mature biofilms formed during 24-h incubations at 35°C were treated for an additional 24h in the same conditions, with antifungal agent concentrations 64 fold higher than the minimum inhibitory concentration (MIC) values determined for the treatment of planktonic cells. Compared to untreated controls **(A-C)**, mature biofilms treated with 32 μg/mL amphotericin B **(D-F)** induced a reduction in the fungal biomass. Treatment with 1,000 μg/mL of fraction F2 **(G-I)** or subfraction F2.4 **(J-L)** induced the formation of elongated blastoconidia cells (arrows in I and L), and treatment with subfraction F2.4 also induced the formation of blastoconidia clusters (arrow in L). Scale bars: 1 mm **(A, D, G** and **J)**, 20 μm **(B, E, H** and **K)**, 10 μm **(C, F, I** and **L)**.

Morphological effects on *C. albicans* biofilms treated with AMB, F2 and F2.4 were evaluated by scanning electron microscopy (SEM) images (Figures 4 and 5). Untreated *C. albicans* biofilms showed a dense network of filamentous form fungi formed for 24 h (biofilm formation assay) (Figure 4A-C) and 48 h (mature biofilm assay) (Figure 5A-C). Treatment with AMB inhibited the fungal filamentation process with most biofilm cells retaining the yeast form (Figure 4D-F; Figure 5D-F). Treatments with fraction F2 or subfraction F2.4 (Figure 4G-L; Figure 5G-L) induced alterations mainly in blastoconidia, with the formation of ‘dumbbell-shaped’ cells (white arrows in Figures 4I, L and 5I, L), suggestive of an effect on the budding process, as previously described for *C. albicans* planktonic cells treated with subfraction F2.4 [7]. Treatment with F2.4 also induced the arising of blastoconidia clusters in biofilms (Figures 4K, L and 5K, L), similarly to previous results of our group for *C. albicans* planktonic cells treated with subfraction F2.4 [7].

Proanthocyanidins are oligomeric flavonoids composed by derivatives of catechin and epicatechin and their gallic acid esters. More complex structures composed of the same polymeric building blocks form the group of condensed tannins. The present work is the first report of the activity of proanthocyanidins polymeric tannins against fungal biofilms. However, other reports have demonstrated the antifungal the activity of flavonoid molecules against planktonic cells of yeast [32,33] and filamentous fungi [34]. Recently, Shahzad et al. [35] described the inhibitory activity of some monomeric polyphenols, including flavonoids, on planktonic cells of *C. albicans* and on *C. albicans* biofilms (at concentrations 78.12 - >1,000 μg/mL and 39.06 - >5,000 μg/mL, respectively).

Subfraction F2.4 was obtained from the aqueous fraction F2 by chromatography using a Sephadex LH-20 column [7], and was identified as a polymeric tannin of molecular weight of 2,114 Da, composed of six monomers of proanthocyanidins (prodelphinidin and prorobinetinidin) and gallic acid residues [5-7]. The degree of polymerization of polyphenols appears to be related to antifungal activity, possibly because the reactivity of tannins with macromolecules (including both proteins and carbohydrates) increases progressively with the degree of polymerization [36,37].

The antifungal properties of *S. adstringens* against planktonic cells of *Candida* spp. and *C. neoformans* have been attributed to this polymeric tannin [7,16]. Our data demonstrate that proantocyanidin polymeric tannin-rich fractions from *S. adstringens* stem bark (fraction F2 and subfraction F2.4) are also active against biofilms of *C. albicans*, likely due to the presence of this proantocyanidins polymeric tannin in these fractions. At safe doses, both F2 and F2.4 inhibited biofilm formation and reduced the metabolic activity of mature biofilm cells. Interestingly, these fractions also reduced the metabolic activity of dispersion cells, indicating that they could inhibit infection dissemination mediated, at least in part, by this cell population. Thus, these polymeric tannins can affect a particularly drug-resistant stage of biofilm development.

## Conclusion

This is the first report of the anti-biofilm activity of proanthocyanidins polymeric tannins from *S. adstringens* stem bark (fraction F2 and subfraction F2.4) and being an important finding, since the development of new and cheaper compounds for the treatment of biofilm-related fungal infections is sorely needed. Therefore, fraction F2 and subfraction F2.4 from *S. adstringens* shows great potential in the search for new therapy against *Candida* infections involving biofilm formation.

## Abbreviations

ECM: Extracellular matrix
SEM: Scanning electron microscopy
MIC: Minimal inhibitory concentration
MFC: Minimal fungicidal concentration
AMB: Amphotericin B
FLC: Fluconazole
^13^C NMR: Carbon nuclear magnetic resonance.
